# Comorbidities of bladder pain syndrome in the context of the HITOP distress category: a systematic review and meta-analysis

**DOI:** 10.1007/s00192-022-05129-1

**Published:** 2022-03-09

**Authors:** Linda Fischer-Grote, Vera Fössing, Martin Aigner, Markus Boeckle, Elisabeth Fehrmann

**Affiliations:** 1 Karl Landsteiner Institute for Outpatient Rehabilitation Research, Vienna, Austria; 2 Scientific Working Group, DOT-Die offene Tür (the open door), Karl Landsteiner University of Health Sciences, Dr.-Karl-Dorrek-Straße 30, 3500 Krems, an der Donau Austria; 3 Department of Psychiatry for Adults, University Hospital Tulln, Tulln, Austria

**Keywords:** Chronic pain, Comorbidities, Interstitial cystitis/bladder pain syndrome (IC/BPS), Meta-analysis, Prevalence, Symptom severity

## Abstract

**Introduction and hypothesis:**

The aim of this systematic review and meta-analysis is, looking at different care settings, to examine prevalence rates of psychological distress-level comorbidities in female interstitial cystitis/bladder pain syndrome (IC/BPS) patients, their impact on Quality of Life (QoL), and the correlation between such comorbidities and symptom severity.

**Methods:**

A systematic literature search according to PRISMA guidelines was conducted in PubMed, PsycInfo, Web of Science, Science Direct, and Google Scholar.

**Results:**

Twenty-nine studies were found that met inclusion criteria. Prevalence rates of depression and anxiety are higher in IC/BPS patients compared to the general population; however, due to a wide array of measurements, statistical comparisons between care settings were only possible in two cases showing mixed results. No studies meeting inclusion criteria exist that examine PTSD and borderline personality disorder, though rates of past traumatic experiences seem to be higher in patients than in healthy controls. Psychological comorbidities of the distress category, especially depression, are found in most studies to be related to symptom severity, also yielding statistically significant associations.

**Conclusions:**

While there is still need for studies focused on some of the comorbidities as well as on different care settings, the data already show that psychological comorbidities of the distress category play an important role in IC/BPS patients regarding suffering, QoL, and symptom severity, thus emphasizing the need for highly specialized interdisciplinary treatment.

## Introduction

In recent years, increasing attention has been paid to the syndrome of interstitial cystitis/bladder pain syndrome (IC/BPS), a chronic disease with the main symptoms of pain, frequency, urgency/pressure, and nocturia [1, 2], which often is accompanied by long-lasting severe suffering [3]. Although the condition is found in both women and men, women are affected at a ratio of 4:1 compared to men. The prevalence rates are estimated at 45 in 100,000 women [4]. The population-based prevalence estimate has been found to range from 2.7 to 6.5 % in American women [3, 5] depending on how specific or sensitive the diagnostic criteria are [6, 7].

Studies suggest that IC/BPS is underdiagnosed and underreported, apparently due in part to imprecise diagnostic criteria. Attempts have been made to establish a more precise classification method of IC/BPS [6], and in the American Urological Association Guidelines a distinct segregation of IC/BPS from similar diseases has been proposed [2]. Especially in men, IC/BPS is underreported [7], and symptoms overlap to a considerable degree with those of chronic prostatitis or chronic pelvic pain syndrome [8].

Similar to other chronic pain conditions, a growing body of literature suggests psychosocial factors play an important role in IC/BPS (e.g., [9]). Relevant psychosocial factors are, among others, maladaptive coping mechanisms, e.g., catastrophizing and fear avoidance [9]. In the treatment of chronic pain, the risk factors for chronification posed by psychological comorbidities are well documented [10]. Several of these, such as depressive symptoms or anxiety, have been found in IC/BPS patients [2, 9]. Not only can mental health problems arise as a response to IC/BPS [2], some evidence hints at common underlying biological factors of IC/BPS and disorders like panic disorder [11, 12]. A recent review reports on varying prevalence rates for different psychological disorders in IC/BPS patients, with these rates for depression ranging from 16 to 70%, for anxiety disorders ranging from 14 to 52%, and for experienced abuse ranging from 25 to 49% [9]. Nevertheless, the review cited does not differentiate between different kinds of prevalence rates and different stages of care, i.e., primary, secondary, and tertiary care. It also fails to consider different psychological comorbidities. This would, however, promote a more differentiated understanding of the syndrome, which in turn could further the development of effective treatment procedures at different stages of care.

In the context of chronic pain, comorbidities like depressive disorder or anxiety disorder have been found to be tightly linked to pain chronicity [13–16]. Additionally, patients with depression frequently report altered pain perception [17], and anxiety has been found to predict pain outcomes [18, 19]. Evidence shows that treatment of either of these comorbidities in chronic pain patients leads to reduced pain intensity and reduced disability though pain [20]. General stress has been found to moderate the experience of pain while continuing stress magnifies pain in a significant number of chronic pain patients [21]. Thus, stress exacerbates the pain experience to the point of making chronic pain itself a stressor (e.g., [22]). In the Hierarchical Taxonomy of Psychopathology (HiTOP), major depressive disorder (MDD), dysthymia, generalized anxiety disorder (GAD), post-traumatic stress disorder, and borderline personality disorder are subsumed under the category “distress” [23], which leads to the hypothesis that these psychological disorders especially may interact with chronic pain and therefore also with IC/BPS. The model implies the underlying modality of distress to be involved in all these comorbidities. Based on the relevance of stress-related symptoms in chronic pain conditions, it can be assumed that all the comorbidities belonging to the distress category enhance symptom severity in IC/BPS. Especially the presence of traumatic experiences might be associated with other psychological comorbidities and symptom severity in IC/BPS patients. It can be hypothesized that traumatic experiences not only lead to an increase in symptom manifestation but also to a higher prevalence of other psychological comorbidities. An overlap between pathways maintaining PTSD as well as chronic pain has already been identified [24]. The review by McKernan et al. gives an overview of studies examining past traumatic experiences in IC/BPS patients and discusses the relevance of PTSD severity for IC/BPS symptoms, while finding a lack of studies on PTSD IC/PBS interactions [9]. Identifying relevant and recent publications dealing with the interplay of PTSD and IC/PBS thus becomes a high priority.

Another important aspect of chronic conditions like IC/BPS is the decrease in quality of life (QoL), a correlation previous reviews discuss especially concerning psychological comorbidities [2, 9]. In patients with depressive symptoms and chronic somatoform pain disorder, a negative correlation with the measurement of QoL has been identified [25], which may be assumed to apply also in IC/BPS. Still, it would be helpful to conduct studies to determine exactly how QoL figures into a comparative analysis of IC/BPS patients with and without depressive symptoms. By definition, one can differentiate between overall QoL and health-related QoL, which focuses on aspects of QoL that are especially relevant in terms of physical or mental health [26, 27]. Sexual dysfunction seems to be a particularly relevant aspect of QoL in the IC/BPS patient population [2].

Based on these earlier findings, some studies are now turning their attention to psychological and interdisciplinary treatments with promising results (e.g., [28]). To help develop personalized, effective treatment methods, a clear, concise knowledge of comorbidity prevalence at different stages of patient care as well as of associations between different comorbidities and psychosocial aspects is of utmost importance. Thus, the aim of this systematic review and meta-analysis is to give a literature overview and meta-analysis regarding the following hypotheses:

- In female IC/BPS patients, the prevalence of psychological comorbidities (depressive disorder, generalized anxiety disorder, and trauma/PTSD) differs depending on the care setting.
- In female IC/BPS patients who have experienced trauma/suffer from PTSD, more additional comorbidities can be found.
- In female IC/BPS patients, symptom severity and QoL is associated with the presence of psychological comorbidities belonging to the HiTOP distress category.

## Materials and methods

### Search strategy

A comprehensive literature search adhering to the Preferred Reporting Items for Systematic Reviews and Meta-Analysis (PRISMA) Framework [29] was conducted in PubMed, PsycInfo, Web of Science, Science Direct, and Google Scholar to locate papers published between January 1995 and June 2020. For detailed search parameters, see Table 1. Google Scholar alerts were enabled to avoid missing accepted articles and articles in preprint. Additional relevant articles were identified by reference search strategy.

**Table 1:** Search parameters used in the literature search divided by database

Database	Search parameters
• PubMed • PsycInfo • Web of Science • Science direct	(“Cystitis, Interstitial” OR interstitial cystitis OR bladder pain syndrome OR mapp network OR chronic prostatitis with chronic pelvic pain syndrome OR chronic pelvic pain syndrome) AND (“Anxiety” OR anxiety OR “Depression” OR “Depressive Disorder” OR depression OR “Sleep Initiation and Maintenance Disorders” OR insomnia OR “Quality of Life” OR “Stress Disorders, Post-Traumatic” OR quality of life OR post-traumatic stress disorders OR “Health Promotion” OR “Psychology” OR psychosocial OR health promotion OR psychology) AND (Clinical Trial OR Comparative Study OR Evaluation Studies OR Meta-Analysis OR Observational Study OR systematic). NOT (drugs OR medications OR prescriptions OR pharmaceuticals)
• Google Scholar	(1) Interstitial cystitis depression OR anxiety OR trauma OR abuse OR “post traumatic stress disorder" (2) Bladder pain syndrome OR depression OR anxiety OR trauma OR “post traumatic stress disorder”

### Study selection process

**Fig. 1:**
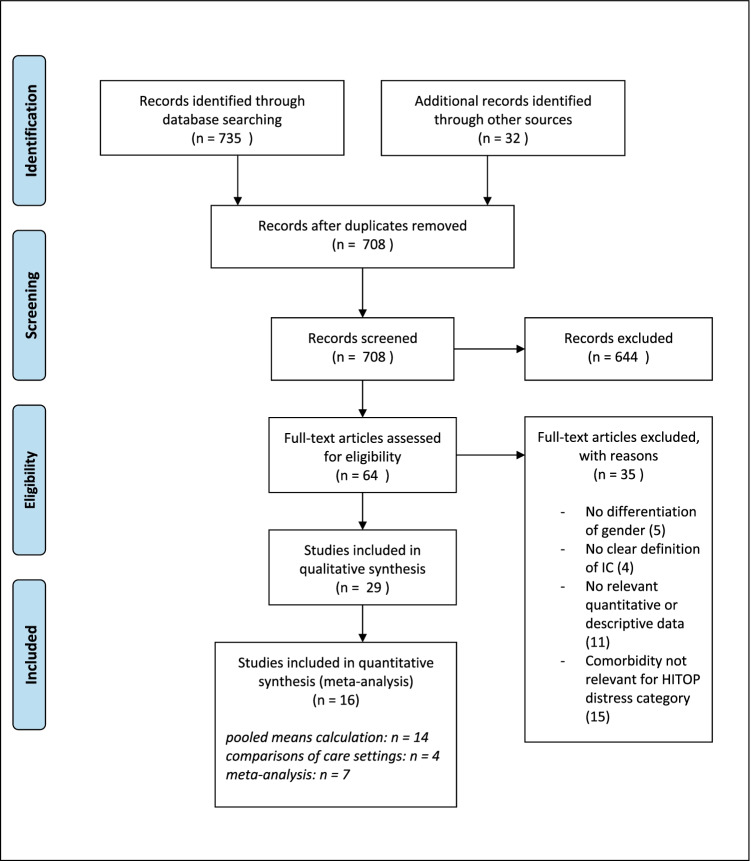
Prisma flow diagram

The title and abstracts were screened for inclusion and exclusion criteria before examining full texts. This was done independently by the authors. For the detailed exclusion process at each stage, see Fig. 1. Inclusion criteria were (1) original studies, (2) published not earlier than 1995 (since at around this time there was an increase in the visit rates related to interstitial cystitis depending on care setting. Before this time little systematic research was conducted including the diagnosis [30]), (3) in peer-reviewed journals, (4) written in English or German, (5) focusing on IC/BPS and psychological comorbidities of the HiTOP distress dimension and quality of life, and (6) focusing on female gender or clearly differentiating between female and male participants to ensure comparability and use of concise diagnostic criteria. To enhance comparability, articles not specifically referring to IC/BPS but instead to chronic pelvic pain, for example, were excluded.

### Statistical analysis

Included studies were assigned to groups according to care settings. Care settings are defined as follows: (1) primary care: first point of consultation for patients: e.g., general practitioners, family physicians, urgent care clinics, health centers; (2) secondary care: e.g., specialists (including clinical psychologists, psychotherapists), hospital care, acute care, general rehabilitation clinics; (3) tertiary care: highly specialized care, facility with personnel and facilities for advanced medical examination and treatment, e.g., university hospitals, tertiary referral centers, specialized rehabilitation clinics [31]. T-tests were applied to compare prevalence rates for different study care settings. In cases of more than one study in one setting or in case of split means for different subgroups in one study, pooled means and standard deviations were calculated using a webtool based on java script that helps calculate pools using sample size, means, and variance [32]. Therefore, studies were grouped based on measures used. Means of studies using the same measures were then pooled. A meta-analysis was conducted to examine the relationship between symptom severity and scores of psychological comorbidities across different studies. To do this, studies examining associations between symptom severity and psychological comorbidities were identified and the relevant specific values extracted and prepared for pooling. In four cases, studies reported coefficients of the correlation of measures of symptom severity (e.g., pain) with psychological comorbidities. In three cases, reported mean differences between high versus low symptom severity subgroups were transformed into correlation coefficients. A webtool [33] was used for this, which computes correlation coefficients from means, standard deviations, and subgroup sizes. For one study [34], the score polarity had to be reversed. The meta-analysis was done with the package “meta” [35], a package for the R environment that includes standard methods for meta-analysis. Effect-size Pearson’s r of all studies was first converted to Fisher’s Z to then determine the weighted average of effect sizes based on r and the sample sizes. According to recommended procedures [36, 37], random effects models were calculated because of variations of sample size, measures, and methodologies between studies. Besides the population effect size and 95% confidence intervals on those estimates, heterogeneity was determined. The “meta” package also produces forest plots. Risk-of-bias assessment for studies included in the meta-analysis was conducted using the Joanna Briggs Institute (JBI) critical appraisal checklist [38], with all studies assessed eligible for inclusion (see Table 2). All statistical analyses were conducted in the R environment for statistical computing [45].

**Table 2:** Risk of bias assessment of studies included in meta-analysis

Study	N	Study type	Fulfilled criteria/overall number of criteria	Overall appraisal	Unclear/not-fulfilled criteria
Chiu et al. (2017) [39]	97	Observational cohort	8 out of 11	Included	- Not applicable: 3 questions regarding follow-up
Ginting et al. (2010) [40]	96	Cross sectional	6 out of 8	Included	- Unclear: information on confounding factors and strategies regarding confounding factors
Lai et al. (2015) [41]	233	Observational cohort	6 out of 11	Included	- Unclear: information on confounding factors and strategies regarding confounding factors *- not applicable: 3 questions regarding follow-up*
Muere et al. (2017) [42]	341	Cross sectional	8 out of 8	Included	
Nickel et al. (2010) [43]	207	Case control	9 out of 10	Included	- Unclear: strategies regarding confounding factor
Tripp et al. (2016) [44]	190	Case control	10 out of 10	Included	
Watkins et al. (2011) [34]	1469	Observational cohort	6 out of 11	Included	- Unclear: information on confounding factors and strategies regarding confounding factors *- not applicable: 3 questions regarding follow-up*

Notes: *N* = number of participants; case control studies were evaluated with the Joanna Briggs Institute (JBI) Critical Appraisal Checklist for Case Control Studies (10 criteria); cross-sectional studies are evaluated with the JBI Critical Appraisal Checklist for Analytical Cross Sectional Studies (8 criteria); observational cohort studies were evaluated with the JBI Critical Appraisal Checklist for Cohort Studies (11 criteria) [37]

## Results

### Sample of included studies

Seven hundred thirty-five articles were found in the initial database search process, and 32 additional studies were identified through reference search strategy. Of these, 59 duplicates had to be removed. Next, 645 articles were excluded since they were not in English or German, were not original articles, were not published in peer-reviewed journals, did not or did not only focus on chronic bladder pain, or did not clearly distinguish between genders. Additionally, articles were only considered if the mentioned outcome variables were explicitly measured. Figure 1 gives a detailed description of the exclusion process.

The final sample comprised 29 articles for the qualitative analysis (see Table 3), of which 16 were included in the quantitative analysis. Of the 29 studies, 13 (44.8%) were carried out in the US, 2 (6.9%) in Canada, 2 more (6.9%) in the US and Canada, as well as 2 each (each 6.9%) in Taiwan, in Taiwan and China, and in Italy and 1 (3.4%) in South Korea. The remaining five studies (17.2%) collected samples from multiple locations: four of them took samples from Canada, the US, Denmark, and India, and one was sourced from the aforementioned countries plus Taiwan. In some cases, country of study implementation was deduced by author affiliation.

**Table 3:** Characteristics and main results of the included studies

Study	Sample size	Gender distribution	Study type	Age mean (SD)	Criteria for diagnosis	Psychological measures	Comorbidity prevalence type	Setting	Main
Cepeda et al. (2019) [46]	N = 3,973,000 general population	50.6% female	Retrospective cohort	50.87 (16.86)	SNOMED criteria	Depression (database)	Point	NR	Being female was risk factor for IC/BPS; individuals with IC/BPS had higher depression incidence than general population; individuals with depression were more likely to develop IC/BPS (0.13% vs. 0.06%); more mood disorders and anxiety in individuals who developed IC/BPS
Chiu et al. (2017) [47]	N = 97 IC/BPS, 43 AC	100% female	Observational cohort	40.6 (10)	AUA guidelines 2010 for IC/BPS (over 6 weeks); other urogenital diseases excluded	Trauma prevalence (BBTS) *, depression (BDI-II), anxiety (BAI), dissociation (TDS)	Anxiety & depression: point; trauma: period	2	IC/BPS reported sig. more physical abuse and childhood trauma by close others than acute cystitis controls; IC/BPS patients had sig. more depression; only IC/BPS patients who experienced childhood trauma perpetrated by close others had sig. more dissociation and anxiety than acute cystitis controls
Chiu et al. (2017) [39]	N = 94 IC/BPS	100% female	Observational cohort	40.6 (10)	Criteria set by authors	Childhood trauma (CTQ), depression (BDI-II), anxiety (BAI)	Anxiety & depression: point; trauma: period	2	Childhood trauma, anxiety, and trauma dissociation associated with increased anesthetic bladder capacity
Chuang et al. (2015) [48]	N = 185 IC/BPS, 370 HC	73% female	Retrospective cohort	46 (16.78)	At least 3 IC/BPS ICD-9 codes from ambulant care visits	ICD codes of depression, anxiety, insomnia	incidence	NR	Individuals with IC/BPS had sig. higher incidence rates in depression, anxiety, and insomnia, were more likely to show healthcare-seeking behavior for mental illness; higher incidence in younger individuals
Chung et al. (2014) [49]	N = 396 IC/BPS, 1980 HC	100% female	Retrospective cohort	47.5 (15.1)	At least 3 ICD-9 IC codes	ICD codes of anxiety disorders	Anxiety: period	2	OR for anxiety disorder after adjustment for medical comorbidities = 4.37
Clemens et al. (2008) [50]	N = 111 IC/BPS, 174 CP/CPPS, 247 HC	IC/BPS patients 100% female	Case control	50 (23–89)	ICD-9 IC/BPS coding	Depression (PHQ-9), anxiety (PHQ-9)	Depression, anxiety: point	3	OR for mental disorder = 8.2; 37% of patients took medication for mental health condition; sig. more IC/BPS patients than controls had mental health diagnosis despite taking medication against them (indicates differences in treatment efficacy)
Crawford et al. (2019) [51]	N = 135 IC/BPS	100% female	Longitudinal observational cohort	52.57 (15.51)	Urologist diagnosis of IC/ BPS	Depressive symptoms (PHQ-9)	Point	3	In IC/BPS patients predicted catastrophizing at 6 months, which predicted pain at 1 year; helplessness as 33 key factors for these relationships
Di Giacomo et al. (2019) [52]c	N = 41 IC/BPS	100% female	Observational cohort	50.17 (11.99)	NR	Depression, anxiety, stress (DASS-21), sexual distress (FSDS), intimacy perception (INTIMACY)	Point	NR	The longer since diagnosis the higher the sexual distress and the higher the intimacy perception; higher sexual distress in turn associated with sig. higher anxiety and depression
El Khoudary et al. (2009) [53]	N = 41 IC/BPS	100% female	Observational cohort	Median age of 38 years (range, 20– 71 years)	NIDDK criteria, negative urine culture, at least 4 points on ICSI	QOL (SF-36)	Point	1	IC/BPS patients score lower on all QoL domains than general female population; symptom severity sig. related to mental health domain
Gardella et al. (2008) [54]	N = 47 IC/BPS, 47 HC	100% female	Case control	38.7 (12)	NIDDK criteria	Sexual functioning(FSFI)	Point	3	IC/BPS patients sig. impacted sexual functioning; IC patients showed higher antidepressant use than controls
Ginting et al. (2010) [40]	N = 96 IC/BPS	100% female	Observational cohort	50.6 (13.8)	Clinical diagnosis of IC/PBlS	QoL (SF-12), depression (CED-D)	Point	3	Depression and mental health QoL domain associated with increased pain
Goldstein et al. (2008) [55]	N = 141 IC/BPS	100% female	Observational cohort	45.9	NIDDK criteria, IC diagosis since at least 6 months	Depression (BDI-II), abuse prevalence (DAQ)	Depression: point, abuse: period and lifetime	3	Sig. higher depression (70%) and abuse prevalence in IC/BPS patients compared to US general population; abuse prevalence highly dependent on type of measure
Kim et al. (2009) [56]	N = 130 IC/BPS, 168 HC	100% female	Observational cohort	74.3	Cutoff ICSI 5 or 7	QOL (KHQ), depression (KGDS)	Point	1	IC/BPS patients had sig. lower QoL and scarcely sig. higher depression than healthy controls; symptom severity correlates with depression and QoL
Konkle et al. (2012) [5]	N = 277 IC/BPS (clinical cohort); N = 3 397 IC/BPS community cohort	100% female	Observational cohort	45.1 (range 18–85)	Referral by specialist	QOL (SF-36)	Point	2	Symptom severity in clinical cohort slightly higher, distress slightly higher in community cohort
Lai et al. (2015) [41]	N = 233 IC/BPS, 191 CP/PPS	IC/BPS patients 100% female	Observational cohort	48.5 (14.7), 39.3 (14.1), 39.6 (14.0)	Criteria set by authors	QOL (SF-12), depression/ anxiety (HADS)	Point	3	Higher symptom severity sig. associated with more depression and worse QoL but not with anxiety
Muere et al. (2018) [42]	N = 341 IC/BPS	100% female	Cross sectional	49.77 (14.49)	Urologist diagnosis of IC/BPS, NIDDK criteria	Depressive symptoms (CES-D)	Point	3	Women who catastrophized showed more illness focused coping, leading to greater sensory and affective pain; this effect was more likely when depressive symptoms were present
Naliboff et al. (2015 [57]	N = 233 IC/BPS, 191 CP/PPS (UCPPS),* 417 HC	55% female	Case control	40.5 (range 19–78)	Criteria set by authors	QOL (SF-36), depression/ anxiety (HADS), trauma prevalence (CTS)	QoL, depression, anxiety: point; trauma: period	3	IC/BPS patients showed sig. increased depression rates, lower QoL and sexual functioning, sig. more early life and adult traumatic experiences; moderate associations between symptom severity and measures of mood
Nickel et al. (2010) [43]	N = 207 IC/BPS, 117 HC	100% female	Case control	49.64 (15.1)	2007 definition of IC/PBS described at the US NIH Urological Chronic Pelvic Pain consensus in Baltimore	Depression (CES-D), anxiety (STAI), sleep (MOSsleep), sexual functioning (FSFI), QoL (SF-12)	Point	3	IC/BPS patients had sig. worse QoL, sleep, depression, anxiety, and sexual functioning than healthy controls; all of these correlated with pain; strong correlation of depression and anxiety with QoL
Nickel et al. (2011) [58]	N = 207 IC/BPS, 117 HC	100% female	Case control	49.64 (15.1)	2007 definition of IC/PBS described at the US NIH Urological Chronic Pelvic Pain consensus in Baltimore	Trauma prevalence (CTES), depression (CES-D), anxiety (STAI), sexual functioning (FSFI), QOL (SF-12)	Depression, anxiety, sexual functioning, QoL: point; trauma: period	3	Non-sig. trend for (1) higher prevalence of rape/molestation in IC/BPS patients than healthy controls, (2) sig. worse pain, depression and physical QoL in patients who did report compared to patients who did not 36 report sexual abuse
Novi et al. (2005) [59]	N = 46 IC/BPS, 46 HC	100% female	Case control	39.2 (11.9)	Newly diagnosed IC/BPS (within the preceding 6 months), cystoscopic findings consistent with NIDDK criteria	Depression (PHQ-9)	Point	2	Symptoms of major depression in 41% of IC/BPS patients compared to 11% of healthy controls; more depression in patients with severe than mild IC/BPS (OR = 10.1); patients with IC/BPS reported sig. worse pain
Ottem et al. (2007) [60]	N = 75 IC/BPS, 22 HC	100% female	Case control	38 (13)	Diagnosis on the basis of a suggestive history and physical examination findings	Sexual functioning (FSFI)	Point	3	IC/BPS patients had sig. impacted sexual functioning in all domains
Peters et al. (2007) [61]	N = 215 IC/BPS, 121 SRIC/ BPS, 464 HC	100% female	Case control	Cases 50.6 (14.8.); controls 50.7 (14.4)	Established IC diagnosis from the investigator clinical database	Trauma prevalence	Trauma: lifetime	3	Higher sexual, physical, and emotional abuse prevalence in IC/BPS patients than controls; mode of questioning (direct interview vs. written questionnaire) impacted responses
Rabin et al. (2000) [62]	N = 74 IC/BPS	100% female	Observational cohort	44.6 (12.4)	In treatment for IC	Depression (CES-D)	Point	3	52.6% of IC/BPS patients demonstrated depressive symptomatology; depression was sig. associated with pain
Rothrock et al. (2002) [63]	N = 65 IC/BPS, 40 HC	100% female	Case control	51.0 (16.1)	NIDDK criteria	QOL (SF-36), depression (BDI, HRSD)	Point	3	IC/BPS patients had sig. worse QoL across all domains, sig. more depression in both measures (prevalence 17%); symptom severity was sig. associated with worse physical and social functioning and mental health but not depression; pain was sig. associated with depression and 4 out of 8 QoL domains
Rothrock et al. (2003) [64]	N = 64	100% female	Observational cohort	50.9 (16.2)	NIDDK criteria	QoL (SF-36), depression (BDI, HRSD)	Point	3	Catastrophizing sig. correlated with increased pain and more depressive symptoms
Seth & Teichman (2008) [65]	N = 119 IC/BPS	100% female	Retrospective case control	40 (13)/37 (12) (without/with history of abuse)	Criteria set by authors	Abuse prevalence, sexual functioning (FSFI)	Sexual functioning: point; abuse: lifetime	NR	Sexual abuse prevalence was 25%; patients who experienced sexual abuse had sig. lower sexual functioning
Tripp et al. (2012) [66]	N = 193 IC/BPS, 117 HC	100% female	Case control	49 (14.9	Diagnostic criteria described in the US NIH Interstitial Cystitis Database Study	QOL (SF-12), depression (CES-D)	Point	3	Sig. more depression in IC/BPS patients than controls; patients with more body pain sites had worse depression; sig. worse QoL
Tripp et al. (2016) [44]	N = 190 IC/BPS, 117 HC	100% female	Case control	Cases 49.20 (14.94); controls 47.83 (13.52)	AUA criteria	Suicidal ideation (PHQ-9/single item), depression (CES-D)	Point	3	23.2% vs. 6% of contols reported suicidal ideation; suicidal ideation sig. associated with pain and depression
Watkins et al. (2011) [34]	N = 1469 IC/BPS	100% female	Observational cohort	46 (range 18-88)	Criteria set by the authors	Depression (PHQ-8), QOL (SF-36)	Point	1	34.8% had probable depression disorder; depression sig. associated with worse mental and physical functioning, pain and symptom severity; patients with depression more likely to seek treatment in primary care and less likely from a specialist compared to IC/BPS patients without depression

Notes: AUA: American Urological Association; CP/CPPS: chronic prostatitis/chronic pelvic pain syndrome; ESSIC: European Society for Study of Interstitial Cystitis; HC = healthy control; MBSR = mindfulness-based stress reduction; N = number of cases; NIDDK: National Institute of Diabetes and Digestive and Kidney Disease; NIH: National Instiutes of Health; NR: not reported; RCT: randomized controlled trial; SD = standard deviation; SNOMED: Systematized Nomenclature of Medicine Clinical Terms; SR-IC/BPS: self-report suggestive of IC/BPS

Regarding the care setting, in 11 cases, no explicit information could be derived from the text. Of these, two used samples from databases. Of the remaining nine studies, authors were contacted, of which seven responded. In the other cases, the kind of setting was determined according to the information available. All in all, 3 samples were from a primary care setting, 5 from a secondary care setting, and 17 from a tertiary care setting, with no information available regarding setting in four cases.

### Prevalence rates for psychological comorbidities depending on treatment setting

#### Major depressive disorder (MDD) and dysthymia

Thirteen out of 29 included studies examined scores and symptoms of depressive disorder in female IC/BPS patients. Pooled means for scores of depressive disorder were calculated across different measures, with average scores showing at least mild depressive symptoms or clinical depression across all included studies (see Table 4).

**Table 4:** Pooled means of psychological variables

Measure	Total *N*	Included studies	Mean (SD)	Clinical cutoff/interpretation
Depression				
BDI-II	234	Chiu et al. (2017) [47] Goldstein et al. (2008) [55]	15.42 (8.5)	> 14 mild depression, > 20 moderate, > 29 severe depression [67]
PHQ-9	157	Novi et al. (2005) [59] Clemens et al. (2008) [50]	8.47 (5.54)	> 5 mild depressive symptoms, > 10 moderate, > 15 moderately-severe, > 20 severe [68]
CES-D	997	Nickel et al. (2011) [58] Nickel et al. (2010) [43] Rabin et al. (2001) [62] Tripp et al. (2016) [44] Muere et al. (2017) [42]	19.04 (13.25)	> 16.0 clinical depression [69], > 19 for clinical depression in chronic pain [70]
Anxiety				
STAI	207	Nickel et al. (2010) [43]	41.82 (15.7)	> 40 cutoff for clinically relevant anxiety [71]
BAI	97	Chiu et al. (2017) [47]	12.59 (9.37)	> 16.00 cutoff for clinically relevant anxiety [72]
Quality of life				
SF-36	1787	Watkins et al. (2011) [34] ElKhoudary et al. (2009) [53] Konkle et al. (2012) [5]	MCS: 44.69 PCS: 39.17	US norms (SDs) for MCS and PCS: 50 (10) [73]
SF-12	536	Nickel et al. (2010) [43] Lai et al. (2015) [41]	MCS: 43.78 (9.56) PCS: 43.16 (9.7)	US norm (SD) for MCS and PCS: 50 (10) [73] Cutoff for 30-day depressive disorder screening: 45.6 [74]
FSFI	279	Nickel et al. (2010) [43] Ottem et al. (2007) [60]	17.78 (10.35)	< 27 optimal cutoff for differentiating between women with and without sexual dysfunction [75]

Notes: BAI = Beck Anxiety Inventory [72]; BDI II = Beck Depression Inventory II [67]; CES-D = Center for Epidemiologic Studies-depression scale [69]; FSFI = female sexual functioning index [76]; *N* = number of cases; PHQ-9 = Patient Health Questionnaire [68]; SD = standard deviation; STAI = State-Trait Anxiety Inventory [71]; SF-12 = 12-Item Short-Form Health Survey [77]; SF-36 = 36-Item Short-Form Health Survey [73].

In six of the studies, the point prevalence was described. In a primary setting, a 34.8% rate of depressive disorders (compared to 5.9–6.7% in the female general population) was found [34]; in a secondary setting a rate of 41% of the IC/BPS patients (compared to 11% in healthy controls) was found [59]; in studies in tertiary settings (*n* = 4), point prevalence rates from 5% of MDD [50], 17% for moderate to severe depressive disorder [63], and from 11% [50] over 52.6% [62] to 70% [55] of depressive symptoms were found. Two statistical comparisons between two studies each were drawn between secondary and tertiary care as measured with two different scoring systems. A comparison of depression scores measured with the Patient Health Questionnaire 9 (PHQ-9) [68] showed a significantly higher depression score in the secondary setting [59] than in the tertiary setting [50], whereas a *t*-test between depression scores measured with the Beck Depression Inventory II (BDI-II) [67] yielded no significant result between secondary [39] and tertiary care [55] (see Table 5).

**Table 5:** Comparison of depression scores in a secondary vs. tertiary setting

Setting	Secondary		Tertiary			
BDI II	Chiu et al. (2017) [39]		Goldstein et al. (2008) [55]			
	M	SD	M	SD	*t*	*p*
	13.65	7.07	14.6	9.2	0.89	0.37
PHQ-9	Novi et al. (2005) [59]		Clemens et al. (2008) [50]			
	M	SD	M	SD		
	15.61	2.81	5.7	5.8	13.73	< 0.001

Notes: BDI II = Beck Depression Inventory II [67]; M = mean; PHQ-9 = Patient Health Questionnaire-9 [68]

*p* < 0.05 indicates a significant result; SD = standard deviation

Regarding incident rates, a study examining comorbidities in men and women with IC/BPS compared to a control group (in primary and secondary settings) found higher incident rates for depressive disorder in the IC/BPS group (101.0 per 10,000 persons per year vs. 42.2 in randomly chosen, matching non-IC/BPS controls) and higher incident rates in women [48]. From another perspective, in a study with women and men, the incidence of IC/BPS was higher in the group of individuals with depressive disorder than in the general population, whereas being female was found to be a risk factor for IC/BPS in both groups alike [46].

Some studies (*n* = 7) did not report prevalence rates per se but reported statistical comparisons of depressive symptoms in IC/BPS patients compared to other groups. In a primary setting, significant differences in depressive disorder were shown between women with IC/BPS with and without sexual distress [52]. More depressive symptoms were found in patients with chronic IC/BPS than in patients with acute cystitis in a secondary setting [47] and higher than in healthy control groups in tertiary settings [43, 50, 63, 66]. Antidepressant use is also higher in patients with BPS than in controls [54].

#### Generalized anxiety disorder

A total of 7 out of 29 studies examined generalized anxiety disorder or symptoms of anxiety in IC/BPS patients. None of the included studies reported prevalence rates, so only comparisons can be reported. No statistical comparisons between settings were possible regarding anxiety, but pooled means were calculated for anxiety scores on differing scales, showing clinically relevant anxiety measured with the State-Trait Anxiety Inventory (STAI) [71], but not with the Beck Anxiety Inventory (BAI) [72] (see Table 4).

In a primary setting, higher anxiety scores were found in IC/BPS patients with sexual distress compared to IC/BPS patients without sexual distress [52]. In a secondary setting, anxiety was significantly higher in IC/BPS patients with a high amount of childhood trauma compared to those with low childhood trauma [39] and also higher in those who experienced childhood trauma perpetuated by close others [47]. In tertiary settings, higher anxiety scores were found in IC/BPS patients compared to controls [43].

Regarding periodic prevalence rates (diagnosis within the last 3 years), one study found a higher occurrence of a prior diagnosis of anxiety disorder in female IC/BPS patients compared to controls (16.16% vs. 3.64%, adjusted OR: 4.37) in a tertiary care setting [49].

Higher incident rates for anxiety were found in men and women with IC/BPS compared to a control group in primary and secondary settings (92.86 per 10,000 persons per year vs. 38.2 in controls) with higher incident rates in women [48]. Another study with men and women found being female to be a risk factor inter alia for the development of IC/BPS, which in turn was related to a higher rate of anxiety [46].

#### Borderline personality disorder

None of the included studies examined the prevalence of borderline personality disorder in IC/BPS patients.

#### Posttraumatic stress disorder and traumatic experiences in the past

Six of 29 included studies examined past traumatic experiences of patients with IC/BPS. Of these studies, none recruited patients in a primary setting, and no statistical comparisons between settings were possible. Compared to healthy controls, women with IC/BPS seem to have experienced more early-life and adult traumatic experiences [57]. A combination of different traumatic experiences was reported as significantly higher than in control cases with 25% in a tertiary setting [61].

Regarding sexual violence, one study found a history of sexual abuse in 25% of women with IC/BPS [65]. In a secondary setting, the periodic prevalence of sexual violence was reported to be 10% in childhood and 9% after the age of 18 [47], whereas in a tertiary setting the periodic prevalence of sexual violence in childhood was reported to be 24% [58]. Lifetime prevalence of sexual violence in tertiary settings ranged from 17.7% [61] to 28% to 36% based on the assessment method [55]. The periodic prevalence for physical violence was reported in 18% of participants under the age of 18 and 25% over the age of 18 in a secondary care setting [47], and 12.7% under the age of 18 in tertiary care [58], whereas a lifetime prevalence of the experience of physical violence was found in 17.2% [61], and up to 31% (based on assessment method) [55] in tertiary care.

Periodic prevalence rates for different traumatic experiences range from 25.1% (extreme illness and parental divorce) to 47.5% (death of family member or friend) in childhood in tertiary care [58] and are reported at a rate of 40% (abuse by close others) in adulthood in secondary care [47], whereas a lifetime prevalence for emotional abuse was calculated at 31.6% in a tertiary care setting [61].

### Associations of psychological trauma with different psychological comorbidities in IC/BPS patients

Of the 29 included studies, 3 took a closer look at relations between traumatic experiences in IC/BPS patients and other psychological comorbidities.

While a study by Nickel et al. [58] found only a trend for differences regarding depressive disorder, anxiety, and QoL in IC/BPS patients with and without sexual abuse before the age of 17, two other studies compared different aspects of traumatization in IC/BPS patients: significantly higher scores were found for depressive disorder and anxiety in patients with childhood trauma compared to those who experienced trauma later in life [39], and significantly higher scores for depressive disorder, anxiety, and dissociative symptoms were also found in patients who had experienced childhood trauma by close others compared to those who had experienced childhood trauma by non-close others [47].

### Symptom severity of IC/BPS in IC/BPS patients regarding comorbidities of the HiTOP distress category

Sixteen studies examined possible interactions of psychological comorbidities with symptom severity of IC/BPS. Symptoms have been found to be more severe in patients with psychological distress in general [56].

#### Symptom severity and measures of mood/depressive disorder

Moderate associations have been found between symptom severity and measures of mood (higher symptom severity going along with worse mood) [57]. Depressive disorder was associated with worser symptoms in general [34, 59], worse functioning [34], increased pain [34, 40], and painful filling and urgency [41]. Patients with more widespread pain have also been shown to be significantly more depressed [66], and depressive disorder was 10.1 times more likely in patients with severe IC/BPS than in patients with mild IC/BPS (48% vs. 13%) [59].

In other studies, only indirect positive associations between symptom severity and depressive disorder influenced by catastrophizing have been found [51, 64], which in turn might be influenced by illness-focused coping [42]. Greater suicidal ideation also seems to be related to greater pain, more depressive symptoms, and more catastrophizing [44]. Another study found greater self-efficacy to be associated with both pain and depressive disorder [62].

#### Symptom severity and symptoms of anxiety

Regarding symptom severity and anxiety, results are mixed as well. In one study, a positive correlation between anesthetic bladder capacity and anxiety was mediated by alexithymia [39]. However, yet another study found no significant differences in point prevalence anxiety scores in relation to IC/BPS symptom severity [41].

#### Symptom severity and traumatic experiences

Three studies examined possible connections between symptom severity and traumatic experiences: One study found positive correlations among anesthetic bladder capacity, dissociative symptoms, and childhood relational trauma, although these correlations were mediated by alexithymia [39]. Patients with sexual trauma seem to have a different symptom presentation with more pain and fewer voiding problems and may have increased central sensitization [65].

#### Symptom severity and quality of life

Mental health-related quality of life was found to be associated with symptom severity [41, 53] and pain [40] in some studies, whereas one study found catastrophizing to be related to pain and worse mental health-related QoL [64].

#### Meta-analysis regarding symptom severity and distress

**Fig. 2:**
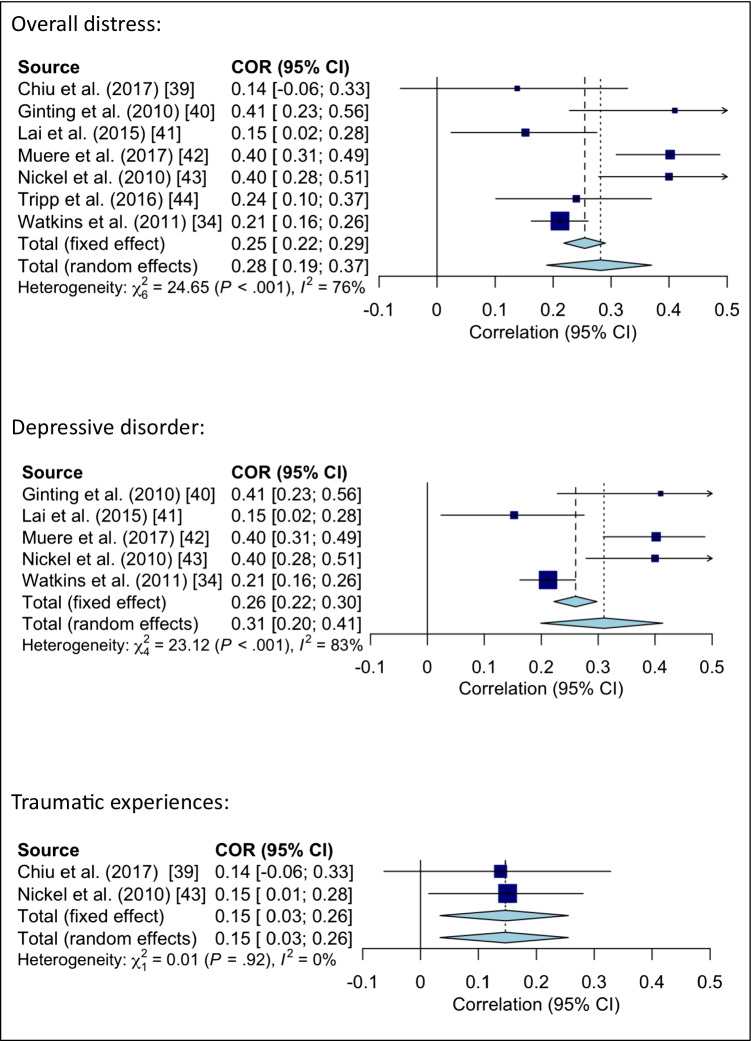
Meta-analysis of symptom severity and overall distress, depressive disorder, and traumatic experiences

A meta-analysis was conducted to examine the strength of the relationship between symptom severity and scores of psychological comorbidities. Based on eligible studies this was done for overall distress and again separately for depressive disorder and traumatic experiences. Random effects models showed significant pooled positive correlations when taking different comorbidities (overall distress = depressive disorder, traumatic experiences, suicidal ideation; see Fig. 2) into account at the same time (r = 0.28, *p* < 0.0001, I^2^ heterogeneity: 75.7%) as well as when only examining depressive disorder (r = 0.31, *p* < 0.0001, I^2^ heterogeneity: 82.7%) or only examining traumatic experiences (r = 0.15, *p* = 0.01) (see Fig. 2 for detailed information on calculations).

### Quality of life in IC/BPS patients in relation to depressive disorder

Seven of 29 included studies examined both QoL and depressive disorder in IC/BPS patients [34, 40, 41, 43, 56, 58, 66]. Pooled means were calculated for QoL measured on two different scales, yielding on average decreased QoL scores in both cases (see Table 4). Three of the studies directly examined the relationship between depressive disorder and QoL in IC/BPS patients and found mild [56] to strong [43] negative correlations between depressive disorder and QoL and significantly lower physical and mental QoL in women with IC/BPS with depressive disorder [34].

## Discussion

The aim of this systematic review and meta-analysis was to take a more differentiated look at studies on prevalence rates of psychological comorbidities of the HiTOP distress category in IC/BPS patients in different treatment settings as well as in terms of symptom severity and quality of life.

Compared to healthy controls or the general population, point prevalence rates and incidence rates of depressive disorder have been uniformly found to be higher in IC/BPS patients, regardless of care setting. Symptoms of anxiety were also higher in IC/BPS patients in terms of point prevalence rates, periodic prevalence rates, and incidence rates, especially in patients with sexual distress [52] and childhood trauma [39, 47]. Several studies reported on a higher number of traumatic experiences in IC/BPS patients [39, 47, 55, 57, 61, 65], whereas no studies examined the prevalence of borderline personality disorder in IC/BPS patients. Several studies showed positive associations between symptom severity and the prevalence of the comorbidities of the HiTOP distress category [34, 39–41, 57, 59, 65, 66] and mental health-related quality of life [40, 41, 53] with only one study yielding no significant result [41]. However, some studies found these associations to be influenced by psychosocial variables [39, 42, 44, 51, 62, 64]. Especially depressive disorder seems to interact with symptom severity and quality of life [34, 43, 56].

All in all, findings suggest that psychological comorbidities of the distress category play an important role in patients with IC/BPS, as comorbidity rates are almost uniformly found to be higher compared to the general population. These findings go along with earlier reviews [2, 9], which also found high rates of comorbidities in IC/BPS patients, with a general understanding of chronic pain in which psychological comorbidities and psychosocial variables are important risk factors in pain chronification (e.g., [10]). This review found comorbidity rates for anxiety disorder and depressive disorder higher compared to controls regardless of setting; however, especially for anxiety disorder, not all treatment setting prevalence rates are reported in the literature. Because of a wide array of measurements used, only two statistical comparisons between settings could be conducted between secondary and tertiary care settings involving depressive disorder with one yielding no significant results and the other hinting at higher depressive symptom rates in secondary than in tertiary care. A possible explanation for more depressive symptoms in secondary care might lie in the higher specialization of the tertiary care setting, which might go along with a higher subjective expectation of patients to receive the right treatment and a feeling of being more comfortable in the tertiary care setting. On the other hand, one might expect a longer period since the onset of the disorder might also result in a higher level of suffering once patients finally reach a tertiary care setting. It has to be noted that in both comparisons only one study each could be included per care setting and measurement. A comparison between larger samples of studies might provide more reliable results. The care setting is of high relevance not only because of the more specialized and comprehensive treatment provided in higher care settings, but also because IC/BPS is still an underdiagnosed disorder in itself [6]. As with other chronic pain conditions, a treatment only focusing on physical symptoms might not be sufficient (e.g., [5, 9]), which highlights the importance of considering prevalence rates in all of the care settings or accelerating the track to tertiary care.

As has been pointed out before, in patients with pain conditions, QoL is associated with accompanying depressive symptoms [25], thus stressing the impact of the psychological strain on pain conditions. In line with this, this review found moderate to high negative correlations between QoL and depression in IC/BPS patients. Moreover, findings on associations between comorbidities of the distress category, especially depressive disorder, and symptom severity highlight the importance of comorbidities of the distress category in IC/BPS. Both pain and depressive symptoms can be viewed as stressors that influence and exacerbate each other resulting in a vicious cycle. It has been suggested that pain as a stressor in itself might exacerbate the perceived intensity of pain and that catastrophizing, i.e., viewing the pain as frightening, might lead to an increased physiological stress response [22, 78]. In turn, an overburdened stress response system might result in less tolerance concerning stress and lead to pain hypersensitivity syndromes [79]. Psychological stress or trauma, on the other hand, seems to increase the likelihood of the occurrence of ongoing pain, as distress, mental suffering [13–15], posttraumatic stress disorders [80, 81], and enhanced numbers of intense childhood or adult adversities have been found to be related to different pain conditions [82, 83]. One study included in this review argued that both depression and IC/BPS show characteristics of inflammatory diseases [46]. Inflammation, depression, and pain may result from cortisol dysfunctions [22]. Pain has also been found to share similarities with fear and anxiety, as an overlap in involved brain areas exists [84]. Aversive past experiences that result in fear and anxiety disorders due to memory traces of overwhelming fear play a role in pain chronification [84, 85]. The association between symptom severity and comorbidities might also be influenced by psychosocial variables [39, 42, 44, 51, 62, 64] like catastrophizing [51, 64], which in itself poses a risk for pain chronification [9].

This review found past traumatic experiences related to symptom severity of IC/BPS to a lower degree than depression, but none of the included studies examined PTBS in female IC/BPS patients. A study done by McKernan et al. [86], including men and women with IC/BPS, found similar rates of traumatic experiences in IC/BPS and other pain conditions; however, a significantly higher prevalence of PTSD in IC/BPS patients underlines the importance of taking a closer look at this disorder in diagnosis and treatment.

The HiTOP [23] has been developed to overcome shortcomings of traditional diagnostic classification systems like the 10th version of the International Classification of Diseases (ICD-10) [87], namely among others co-occurrences of disorders, imprecise boundaries of disorders, and heterogeneity within disorders. To do that, it applies a more dimensional, hierarchical approach, which combines related symptoms and arranges co-occurring syndromes [88]. Considering the findings reported in this review, IC/BPS could also be a cluster of symptoms related to the HiTOP distress category, depicting some of the diagnostic characteristics of distress that occur among the other syndromes of the distress category. However, IC/BPS might also show similarities to the disorders subsumed under the HiTOP category of somatoform disorders, and more research might be needed to examine this interrelatedness more closely. The relative inability to clearly distinguish somatoform disorders from the internalizing spectrum which subsumes distress is even an issue raised during the validation of the HiTOP [88]. Be that as it may, the relevance of psychological interventions/psychotherapy as a very important part in the therapy of IC/BPS becomes clear as early as possible, i.e., the earliest stage possible of chronification as possible, to prevent further chronification and the development of more comorbidities. As this review shows the positive relation of symptom severity and the occurrence of comorbidities, the severity of symptoms might also be an indicator of the need for psychological therapy. This also shows the need to alert professionals even in primary care settings so that psychological therapies can be facilitated early on.

### Limitations and implications for future research

While this systematic review and meta-analysis sets out to give a clear picture of prevalence rates of distress comorbidities for different care settings, distinguishable figures for all disorders and for all types of care settings are not to be found in the literature, and calculations that were possible could only be made with a small number of studies, whereas larger numbers of included studies for calculations would possibly lead to a greater generalizability. Since only few studies were eligible for the pooling of statistical characteristics, possible confounding factors such as age could not be considered, which might be an interesting aspect for future research. Due to the kind of included studies, no certain inference is possible regarding direction of associations between the comorbidities of relevance and IC/BPS or causality. More longitudinal studies would be needed to examine this aspect. For borderline personality disorders, no studies could be found that matched inclusion criteria. To increase comparability and to ensure a more concise definition of the syndrome in question, this review only included studies on female IC/BPS patients or studies from which results for men and women could be clearly differentiated. Nevertheless, due to this decision some relevant studies might not have been included, as, for example, the only study on PTSD symptoms [86].

### Conclusion

This systematic review and meta-analysis has provided some important insight into findings on prevalence rates of psychological comorbidities of the HiTOP distress category as well as on associations of QoL in IC/BPS patients; in doing so, it has integrated different comorbidities in relation to IC/BPS instead of just looking at them one at a time. Although more studies are needed in the areas of care settings and conditions like borderline personality disorder and PTSD, this review has exposed the interrelatedness of psychological distress and IC/BPS in the vicious cycle of distress and chronic pain. By taking a differentiated look at care settings, this report has set in relief the need for interdisciplinary treatments of IC/BPS that also focus on the psychological comorbidities. It seems clear that treating one without the other might not be sufficient in alleviating suffering in IC/BPS patients. This highlights the urgent need for complex, specified therapies and psychological interventions in the treatment of IC/BPS patients as early as possible to slow chronification processes and to prevent the development of additional comorbidities, especially, but not limited to, in patients with a high symptom burden of IC/BPS.
